# Notices, Accumulated Odds and Ends, Etc.

**Published:** 1860-10

**Authors:** 


### Notices, Accumulated Odds and Ends, etc.

ONE of the very best family religious newspapers in the United States, abounding in sterling instructive practical matter, is the *Presbyterian Expositor* of Chicago, N. L. Rice, D.D., editor; the "Upper House" must have a hand in it.

THE WORLD, a daily paper in New-York City, larger than either the *Herald*, *Tribune*, or *Times*, attained, in less than a month a *bona-fide* circulation of over thirty thousand copies daily. Its price is one half that of either of the papers named, being one cent each, or three dollars a year; the weekly edition is two dollars. It is edited by Spaulding, formerly of the *Courier and Enquirer*, and Cummings, who made the *Philadelphia Bulletin* what it is. It is conducted on Christian principles, being the steady and consistent friend of religion, of the Bible and of the Sabbath-day, never admitting any thing into its columns which parents may not read to their children, or which a gentleman might not read to a lady. Its commercial and telegraphic reports are as extensive as those of any paper in the city. It aims to give such items of news as are known to be true; while its special foreign correspondents write from the places designated, and communicate actual facts rather than theories and surmises. Multitudes regarded a "religious daily," as it was rather tauntingly called, the merest chimera; but Christian people and reflecting business men and sterling citizens have given it a welcome such as no other daily paper, the world over, ever had before; already its editorials are largely quoted. It is independent in all things, neutral in nothing; and conducted thus far on the basis of a liberal but decided religious sentiment. We have said thus much without having any personal acquaintance with the editors; because a paper with a *uniformly* healthful tone promotes healthful morals in the families which it visits; and good morals are the safeguards against vice, intemperance, disease, crime, and premature death.

One of the penalties of greatness is, that its sentiments are persistently misunderstood or wilfully misrepresented. The editor of the JOURNAL OF HEALTH!! has the reputation of being a very plain-spoken individual, and yet he is represented from Dan to Beersheba as being opposed to early rising. Whatever his theories may be, his practice is to be ready to read, write, prescribe, or take a fee as soon in the morning, winter or summer, as a man can use his eyes conveniently without "specs," for he is not old enough to need them yet. The renowned Andrew Jackson Davis, editor of the *Herald of Progress*, says: "Dr. Hall's philosophy of late to bed and late to rise, will meet the wishes of the night-loving population of gas-lit cities, and of those who indulge in midnight debaucheries, voluptuous indulgencies, late novel-reading, and intemperance. But," says the "seer," in the same article, "we do enthusiastically urge the practice of early to bed and early to rise, with a substantial meal to start upon the duties of the day, as the most rational and harmonizing life for intelligent human beings to live." It would have been almost impossible for him to have expressed our own views more precisely, unless our identical words had been employed, (see page 116, Vol. 7, 1860:) "Breakfast should be eaten in the morning before leaving the house for exercise or labor." That seems pretty plain!

Again, in our article on sleeping, (see *Health Tract*, No. 25 :) " Go to bed at a regular early hour, not later than ten, and get up as soon as you wake of yourself in the morning." Now, if any body can frame a plainer or more explicit expression of sentiment about early rising, we will give such a person credit for being a good old Anglo-Saxon scholar. Now, friend Davis, we are going to give you a dig under the fifth rib, and you won't forget it even beyond the boundary-line of the grave. Do you write as carelessly about the Bible and the received Christian sentiment of the age as you have done of us and our JOURNAL? Have you formed your religious opinions on such a loose reading of the Scriptures as of our article? The character, the quality of a man's mind "runs" the same, on whatever subject it expends itself, and it is essentially the same in all the phases of life. The JOURNAL was plain; you read it, or you did not read it; then represented it as advocating precisely what it did not advocate, and belabored the Editor accordingly. You read the Bible, or you did not read it, and then, in your issue of July 14, you commend a book, and offer it for sale as suitable "for the thousands who have been misled by mistaken believers in Bible infallibility." Perhaps the same looseness of investigation has led you to make a standing butt of the clergy as a class, using towards them epithets of the most degrading character, and then turn round and expend all your sympathies in behalf of the New-Jersey wife-murderer, who poisoned his victim while caressing her on his knee. The same kind of an examination of the proprieties of things leads you, in an article on Sunday Morality, July 14, to quote commendingly the sentiments of a Sunday paper, which denies the right of any government to make the Sabbath different from any other day as regards restrictions from labor or enjoyment. If you strive to destroy the Bible, which advocates every where purity and justice; if you strive to destroy the character of the clergy, who, as a class, are the most learned, the most blameless, and the most useful men in the world, and plead for the life of the deliberate murderer of his own young, confiding wife; if you strive to secure for the ignorant, the idle, the degraded, and the drunken such an observance of the Sabbath-day as may suit them, contrary as it is to the law of the land, while it interferes with the enjoyment of that peace and quiet which is preferred by the educated, the refined, the industrious, and the temperate, it may reasonably be supposed that you have arrived at these ends by looking at the Bible, at the clergy, at the justness of the laws of the land, and the Sabbath-day, as you did at our article on Early Rising, without any careful examination, and an utter recklessness of the results of making untruthful statements. These remarks are pertinent to this publication; for in proportion as communities disregard Bible teachings, and desecrate the Sabbath-day, in the same ratio do they become degraded, vicious, beastly, running into all kinds of animal indulgences, until the body becomes literally rotten in its rioting, perishing before its prime, leaving its generations withered and diseased at the root and blasted in the bud. That you may re-read the Bible, and give it the candid examination of an ingenuous, unprejudiced, and philosophical mind, is our earnest desire.

One of the leading publishing houses of this city, the Mason Brothers, Nos. 5 and 7 Mercer street, have issued the "Avoidable Causes of Disease," by John Ellis, M.D., 400 pp. 12mo. It is highly commended by the editors of several religious newspapers. We are persuaded that they never could have read the book, any more than the excellent publishers; or any more than we could have read one of the advertisements in the September JOURNAL OF HEALTH, which contains the biggest lie ever put in print. The advertiser declares that his "dietetic saleratus is as harmless to the stomach as flour itself." Now, we will be charitable all round, and suppose that the advertiser employed some body of more impudence than wisdom to write his advertisements, as Brandreth and the celebrated Medicated Inhalation man used to do. Dr. Ellis teaches that costive persons generally live to grow old. He quotes freely from the publications of Fowler & Wells, and uses Catherine Beecher and Horace Greeley with liberal scissors. In a preface of seventeen pages, he often speaks of our "Heavenly Father," of "love to man," "Christian," "Divine command," etc., concluding the volume by suggesting that church-members had a great deal better give their money to the "heathen at home" than to those ten thousand miles away. Such a novel idea! So generous to dictate how people should spend their own money! Such admirable taste, so democratic, to advise the religious and the rich that they had better help the poor around them more, and dress less expensively, live in plainer houses, and worship in plainer churches. All this, and a good deal more of the same sort, in a book purporting to treat of the "avoidable causes of disease!" It really seems to us that it is becoming more and more impossible to take up a book or a newspaper or go to a lecture without being nauseated or outraged, according to the taste of the hearer, with insufferably stale nonsense in ridicule of the rich and the religious. What are we coming to?

Laws of Life.—Sometimes assertions are made, and earnestly believed, too, by those who make them, which are so supremely absurd that one feels as if it would be an utterly hopeless task to correct the error, among which are as follows: A Hydropathist Editor says, in a published lecture, that eating the meat of animals fatted for the market is a deadly poison. Then, again, that vaccination occasions a terrible loss of life; and that Dr. Jenner's name will yet "be mentioned with cursing and bitterness;" because vaccination has introduced the seeds of scrofula very generally; but that it is more easily done by eating the meat of fatted animals. The time when Jenner is to be "cursed," is when it will be discovered that vaccination causes scrofula; but inasmuch as the same thing is done by eating the meat of fatted animals, it will be difficult, between the two causes of scrofula, to decide which is which. We advise the editor not to wait, else he may have a chance of getting as gray as a thousand-year-old rat before he can safely "curse Jenner" or roast beef.

What is the reason that the cold-water people are so full of cursing and bitterness? See how another Water-Cure journal ventilates itself. In the August Journal of Health we advised the free use of the tomato as a health-promoting article of diet, premising that it had medicinal effects, by reason of the seed acting as the seeds of grapes, white mustard, and figs are generally supposed to do by educated physicians the world over, that is, by keeping the bowels free, as multitudes have found to be the case. But our theory of the "*quo modo*" of their action is abhorrent to the mind of the Editor of the *New-York Water-Cure Monthly*, and he forthwith plunges at us with the savageness of a meat-axe, and talks about "pulverized granite, pounded glass, epsom salts, tin filings, cayenne pepper, nettle-stings, thorns, thistles, etc.," being as good as tomatoes for the purpose and manner advocated by us. And, as proof, he asks a question, and makes a statement. The question is: "Why can not the flippant editor see deep enough into physiology to understand" better? The statement is, that he has not used any of the above articles in fifteen years, and is in good health. We verily thought that cold water had a clarifying effect, but we presume that it does not extend to the brain.

What is the reason that "isms" go in droves? There seems to be a kind of brotherhood among the whole of them. There is one element which seems to be a connecting link between them all, which makes them conglomerate; perhaps it is Spaulding's glue! No, it is not that. We must search lower down than the hoof of a beast to get the fundamental principles of affinity between Water-Curers, Phrenologists, Free-Lovers, and Harmonialists. Just see how an admirer of Seer Davis meets the apparently harmless suggestion of ours, that it is rather better for persons to eat their breakfast before they go out to work or exercise. The man does it bravely, too; he is not afraid or ashamed of his birth-place or his name: "Horace Steele, Painesville, Ohio, July, 1860." "It is deplorable that any man who claims to publish a JOURNAL OF HEALTH should sink himself so low as to become a panderer to vice, by prescribing the means by which its practice may be continued. You gave Dr. Hall a severe rebuke, but not any more so than he deserves." Then the Seer Davis comments: "We sincerely thank our elder Brother Horace, in behalf of the cause of human redemption from disease, for his straight-out and truthful testimony in favor of early rising." What is the connection between our advice, that a man should eat his breakfast before he went to work, and "pandering to vice," must be left to some highly imaginative individual, some man whose appropriate phrenological bump is a mile high, or whose "frankness" is a mile deep. The "seer," however, goes on to say, that he, too, had advised that something should be eaten before going out of mornings, at least an "orange," something to stay the stomach; but he says he only meant that it was necessary for the "poor Irish," "widow women with large families," "poor seamstresses," and the "debilitated." Why, Andrew! how came you to make such a fool of yourself? You are as great on "Harmonies" as the man on "Fits," and have almost as much of the milk of human kindness in you as the Water-Cure people. But we excuse you in part; these things were written in the dog-days, and that divinity was in the ascendant; hence the snapping and the snarling. "Just so."

Let us make a clean sweep of the rubbish while we are at it. In the same "dog-days," the *United States Journal*, in reference to our recommendation that berries and fruits, ripe, fresh, and perfect, should be freely used in summer, as being at once nutritious, agreeable, and healthful; and in the same direction that the acidity of butter-milk, clabber, and the like, led some communities to use them largely, tilts at us in manner, form, and words, to wit: "It is really surprising how attractive learned nonsense is to a great many people." Then the editor blazes away at the *Tribune* for copying it, concluding thus: "A bundle of greater absurdities we have seldom seen put together. If Dr. Hall can eat butter-milk, suet, alum, acid, gooseberries, currants, and vinegar, congratulate him on having a strong stomach! but don't undertake to follow his example."

We invite the reader to put two things together. In that same paper there is a leading editorial, and a very long advertisement. The leader talks about *Uncle Tom's Cabin*, and the advertisement speaks of a medicine, which has wonderful properties in imparting strength to the debilitated, and health to all who need it. This same mixture is in quart-bottles that don't hold a quart, at two dollars each; to be taken in doses which will make a bottle last a week; but if that is not enough, take a whole bottle. The medicine is essentially iron-rust and molasses; iron is cheap, and so is molasses. Let us see.

There is a spoonful of iron-rust in a bottle, costing a quarter of a mill, the bottle six cents, and the molasses twelve cents a gallon, (New-Orleans,) or three more cents for the short quart, total nine cents and one third of a mill. The former publishers of *Uncle Tom's Cabin* having failed to place themselves on the "retired" list by publishing fiction, have changed the phase of their tactics, and hope to replenish an exhausted treasury, by dealing in the more substantial articles of iron and molasses; and as one kind of "progress" is uniformly downwards, we may find the same enterprising gentlemen, in due time, "fetching up" in another harbor, loading ebony "for a market." Now as the water-cure people and patent medicine dealers make common warfare against the JOURNAL OF HEALTH whose fundamental principle is never to advise a dose of medicine, and whose object is to teach people how to live healthily by the wise employment of food and exercise, it might seem to some a legitimate inference, that the motives of these parties were quite as pure as those of Alexander the coppersmith; and that they are in trepidation, that if the principles of the JOURNAL prevail, there will be no body to sell their medicines to, no body to be sloshed with cold water, and they will have to go to plowing, which wholesome occupation they have sufficient capacity for, and which they ought not to have ever left; a mistake, as unfortunate for themselves as for society. Now Andrew! be "harmonial," and ye watery spirits who preside at Dansville and Laight street, keep as cool as the divinity you worship, write us a laughing reply, and thus get rid of your "black bile;" it is almost as certain as a good dose of calomel, to rid you of anger, wrath, malice, and all uncharitableness, and you will sleep better for a month afterwards. Wonder if the cold-water people ever do laugh? One would not think so from their writings. By the general tenor of their monthlies, we are inclined to conclude that their sole articles of food are "barks" and snapping-turtles, with alum-water and persimmon-cider as beverages; they are so puckered up, so constringed are they, that the mollifying juices of human kindness seem never, by any chance, to exude from their rhinoscerostic hides or hearts.

MIASM.—Of all our exchanges, as far as yet observed, only one has shown a proper appreciation of the value of our article in the September number.

The editor of the CONGREGATIONAL JOURNAL at Concord, New-Hampshire, says: "The first two articles are among the best that ever appeared in the JOURNAL OF HEALTH. That on Miasm is worth twice the subscription-price." We ourselves add that the article deserves to be framed in every household; its principles are of *vital* importance, literally, to every man who wishes to rent or buy or build in any part of the globe. These principles are as eternal as the laws of matter; they can never change, and our highest health and safety depend on our wisely adapting ourselves to them.

THROUGH every change and crisis and revolution in the civil, political, and financial world he is safe who abhors debt.

THE ANÆSTHETIC INHALER, invented by H. G. Luther, Dentist, 42 Great Jones street, New-York, heavily plated with silver, price five dollars, is pronounced by Drs. Carnochan, Francis, and Mott, to be superior to any thing of the kind yet devised in France, England, or America.

PHOTOGRAPHIC ALBUM, by Messrs. Anderson & Archer, 22 and 24 Franklin street, New-York, makes one of the most beautiful and interesting centre-table ornaments; it is arranged to receive the photographs of kindred and friends, to be looked at by turning over a leaf, instead of the cumbersome opening of cases. The leaves are so attached to the back with strong linen, that apparently they would not be displaced, and could not be torn out, in the ordinary handling of a century. In these days of interchanging photographs, this is one of "hits" of the times; there ought to be one on every drawing-room table.

STUDYING OUT OF SCHOOL-HOURS.—The wise and thoughtful care of human health and happiness and life has led our excellent Superintendent of the Public Schools of the city, to recommend to the Board of Education the abolition of study out of school-hours absolutely in the primary departments, and to curtail them largely as to the more advanced scholars. It is earnestly hoped that the Board will follow the humane example set in other cities. To be kept at study from nine in the morning until three in the afternoon, thus involving an absence from home of seven hours every day, six of which are employed in severe mental application, with a few minutes' intermission now and then, is nothing short of a barbarity worthy of the ignorance of the middle ages, of the days of Salem witchery and ordeal of fire and water; but when in addition to this, lessons are given to be learned at home, which require the less bright scholars to rob themselves of necessary sleep in order to be able to learn them properly, and to which they are stimulated by goadings more imperative than the lash, is an enormity which is inexcusable and inhuman. We hope that the *Press* of this city, following the example of *The World*, will urge this reform with pertinacity and power.

HOW TO GET GOOD COAL FOR THE COMING WINTER.—Buy it now from dealers who ask the highest price, and save it by throwing your furnaces into the river, and use the Low-Down Grate in your parlors and sitting-rooms, and thus have some kind of cheerfulness about your homes. In addition, use the Ash-Sifter sold at 63 East-Sixteenth street, by William E. Jones, Esq.; it is undoubtedly the best ever yet invented. Price, four dollars.

SIGHT-SEEING.—One of the most interesting and pleasurable sights in New-York, is the Aquaria at Barnum's Museum. We took some ladies there the other day from Philadelphia. One of them exclaimed: "I could remain here a week." To see how fish deport themselves at the bottom of the ocean, is well worth a visit to the Museum, were there not a thousand other objects of interest there, and all for twenty-five cents. The Museum has never presented so many attractive features as since Mr. Barnum's reïnstatement.

ODD NUMBERS of HALL'S JOURNAL OF HEALTH sent to the address of Dr. W. W. Hall, New-York, post-paid, one cent each, will be received in payment from subscribers, at six cents each, for any of the Editor's publications, or for new subscriptions to the JOURNAL or the FIRESIDE MONTHLY, if sent during October of the present year.

MUSIC.—One thing at a time, and that well, is the admirable motto at the Normal Academy of Music at Salem, Ct., which has been founded by the enterprise and energy of the Hon. Oramel Whittlesey. Music only is taught there, vocal and instrumental; and, while on so charming a subject, we may appropriately add a paragraph from the FIRESIDE MONTHLY for September, on the subject of MUSIC FOR OUR DAUGHTERS: "Southerners who are now flocking to our city, will find it to their interest to call at Worcester's spacious Piano Establishment, on Fourteenth street, corner of Third Avenue, one of the very oldest Houses in New-York. In all the financial crises of the country, it has never known a 'suspension,' or a 'removal'; thus indicating a thrift, which is only known to men who always make the best instruments, and thus secure the steady patronage of wealthy families, which, from its extent, enables the proprietor to sell a better Piano at a lower price than can elsewhere be had, being more especially adapted to withstand the effects of a warm and moist climate."

### NOTICES, ACCUMULATED ODDS AND ENDS, ETC.

To Parents living in the vicinity of 339 Fourth Avenue, Twenty-fifth street, we heartily commend Mrs. M'Millan, as a teacher of children; her terms are from five to ten dollars a quarter. Her excellent husband lost his health while a foreign missionary, and she now seeks to provide for her interesting family by teaching school. We join in the commendation of Mrs. Erastus Brooks and other ladies, the wives of some of our first citizens, as to Mrs. M.'s ability and fidelity.

An enthusiastic Virginian thus piles up the praises with accumulative power of the article in the September Journal on "'Household Slaveries,' every word of which should be written in letters of gold on the four walls of every room in every man's house in the world, and read daily."

Mrs. Steel's Preparatory School for Boys and Girls at 70 Irving Place, is worthy of neighborhood patronage, supported as it is by such prominent ladies as Mrs. Dr. Doremus, Clark, Bishop, Ward, Schott, and Clapp, all living in and around Grammercy Park, Union Square, and Irving Place, called the Regal Region of New-York.

Liquid Glue.—The French make it thus: In a wide-mouthed bottle dissolve eight ounces of best glue in a half-pint of water, by setting it in a vessel of water and heating it until dissolved. Then add slowly, constantly stirring, two and a half ounces of strong aquafortis, (nitric acid.) Keep it well-corked, and it will be ready for use. This is the "Celebrated Prepared Glue," of which we hear so much.

The Fireside Monthly.—The character of its articles may be known from the headings of the first six numbers; see one of the advertising pages. It aims to exclude fiction, trash, and prosy treatises. No article is ever admitted adverse to the Bible, to Religion, or the Sabbath-day: it will abound in short practical pieces, written by persons of acknowledged ability, such as may be read with profit at any time, in any place, and to any company. Some of the sweetest pieces of poetry in the English language, will be found in the September number. The June number contains thirty-six one-page Health Tracts on a great variety of subjects, succinct and practical, a vade mecum of Hygiene, which every man and woman, boy and girl, worker and idler, rich and poor in the land ought to read; sent post-paid by the Editor for fifteen cents. It is one dollar and a half a year; afforded to subscribers to Hall's Journal of Health for two dollars a year for both monthlies. Back-numbers of both can be furnished to any desired extent.

Harness a horse if you want him to leave a burning building quickly.

The *British and Foreign Medical Chirurgical Review*, republished by the Messrs. Woods, 389 Broadway, at three dollars a year is enriched by several original communications on insanity, chorea, ethnology, etc., with a handsome notice of the work of our countryman, Austin Flint, M.D., with a great variety of miscellaneous medical matter. The American Phrenological Journal for August is of unusual interest; it contains a handsome portrait of Townsend Harris, Esq., our able and efficient and honored Minister to Japan, and several pages of an admirable lecture on Physical Culture, by Henry Ward Beecher. The Water-Cure Journal for August, 308 Broadway, 10 cents, has a valuable article on the Feet and Hands, their pains and penalties, by Dr. Trall, in reference to corns, bunions, callosities, etc. It is inexplicable to us how the American Agriculturist can give for one dollar a year thirty quarto pages monthly, so much valuable, practical and varied reading matter, a healthful moral tone always pervading its clean white pages. No farmer's house would fail to be benefited by such a publication. Our old acquaintance, The

COUNTRY GENTLEMAN, of Albany, issued weekly, is still the favorite of thousands of thrifty husbandmen; two dollars a year, vol. 16. THE METHODIST, two dollars a year, 7 Beekman street, New-York, we commend heartily to every conservative member of the Methodist Church. With John A. Gray as Printer, Lemuel Bangs Publisher, and Drs. Crook and McClintock Editors, it could not but merit the high praise of the *New-York Observer:* "Beautiful in its dress, rich, varied and attractive in its contents, giving fine promise of vigorous life, and great usefulness. Conservative, wholesome, alive, and earnest, edited with taste, tact, and ability, it must make its mark on the Church, and we hope it will become a power in the world."

SPECIAL NOTICE.—All subscriptions to HALL'S JOURNAL OF HEALTH begin with the January number of each year, for convenience of binding and index. Those who wish to subscribe now, can send one dollar for 1861, vol. 8, and at the rate of eight cents for each number up to December next inclusive, in postage-stamps.

OLD NUMBERS: six cents each, in any of our publications, will be allowed for any of the old numbers of HALL'S JOURNAL OF HEALTH sent post-paid by mail, (one cent each,) or by private hand to "Dr. W. W. Hall, New-York," any time during the month of October, 1860. The six vols. of HALL'S JOURNAL OF HEALTH, bound two vols. in one, with a copper-plate engraving of the editor's portrait in 1847, and the three vols. of BRONCHITIS, CONSUMPTION, and HEALTH AND DISEASE, bound uniformly in morocco in the best style, are sold at 42 Irving Place only; price, Twelve Dollars. The *Presbyterian Historical Almanac* for 1861, by Joseph M. Wilson, Philadelphia, will soon be ready, and ought by all means to be supplied to every clergyman of the denomination who belongs to the Old School Presbyerian Church. Price, One Dollar. Stereotyped.

JOURNAL OF HUMAN SCIENCE, vol. 1, No. 1, Cincinnati, O., 32 pp. 8vo. two dollars a year, edited by Wm. Byrd Powell, M.D., a medical scholar and writer of ability. This number contains a large amount of very curious and instructive information on the subject of the Temperaments, which is handled in a masterly manner.

### CONTENTS OF HALL'S JOURNAL OF HEALTH FOR OCTOBER.

(New-York, $1 a year.)

